# Systemic Administration of Induced Neural Stem Cells Regulates Complement Activation in Mouse Closed Head Injury Models

**DOI:** 10.1038/srep45989

**Published:** 2017-04-06

**Authors:** Mou Gao, Qin Dong, Hui Yao, Yingzhou Lu, Xinchao Ji, Mingming Zou, Zhijun Yang, Minhui Xu, Ruxiang Xu

**Affiliations:** 1 Department of Neurosurgery, The Third Affiliated Hospital of The Third Military Medical University, Chongqing 400042, China; 2 Affiliated Bayi Brain Hospital, P.L.A Army General Hospital, Beijing 100700, China; 3 Department of Neurology, Fu Xing Hospital, Capital Medical University, Beijing 100038, China; 4 Department of Obstetrics, Fu Xing Hospital, Capital Medical University, Beijing 100038, China

## Abstract

Complement activation plays important roles in the pathogenesis of central nervous system (CNS) diseases. Patients face neurological disorders due to the development of complement activation, which contributes to cell apoptosis, brain edema, blood-brain barrier dysfunction and inflammatory infiltration. We previously reported that induced neural stem cells (iNSCs) can promote neurological functional recovery in closed head injury (CHI) animals. Remarkably, we discovered that local iNSC grafts have the potential to modulate CNS inflammation post-CHI. In this study, we aimed to explore the role of systemically delivered iNSCs in complement activation following CNS injury. Our data showed that iNSC grafts decreased the levels of sera C3a and C5a and down-regulated the expression of C3d, C9, active Caspase-3 and Bax in the brain, kidney and lung tissues of CHI mice. Furthermore, iNSC grafts decreased the levels of C3d^+^/NeuN^+^, C5b-9^+^/NeuN^+^, C3d^+^/Map2^+^ and C5b-9^+^/Map2^+^ neurons in the injured cortices of CHI mice. Subsequently, we explored the mechanisms underlying these effects. With flow cytometry analysis, we observed a dramatic increase in complement receptor type 1-related protein y (Crry) expression in iNSCs after CHI mouse serum treatment. Moreover, both *in vitro* and *in vivo* loss-of-function studies revealed that iNSCs could modulate complement activation via Crry expression.

The complement system, an important regulator of immune responses, consists of a large number of proteins that are mainly synthesized in live cells and circulate in the blood^1^. Moreover, complement components can also be produced by neurons, astrocytes and microglial cells in the brain^2^,^3^. When activated, the complement cascade leads to the production of anaphylatoxins (C3a and C5a) that mediate inflammation and the assembly of membrane attack complexes (C5b-9) that mediate cytolysis^3^,^4^. There is growing evidence that complement activation plays important roles in the pathogenesis of central nervous system (CNS) diseases^5^,^6^. Patients face neurological disorders due to the development of complement activation, which contributes to cell apoptosis, brain edema, blood-brain barrier (BBB) dysfunction and inflammatory infiltration in various pathological conditions, including infection, ischaemia, hypoxia and trauma^5^,^7^,^8^.

Substantial evidence also suggests that the levels of complement factors involved in inflammation-induced secondary brain damage are increased in brain injury^9^,^10^. In human traumatic head injury patients, complement proteins C1q, C3b, C3d and C5b-9 were increased in neurons in the penumbra region of the contusion^9^. Additionally, elevated levels of C3 and factor B were detected in the cerebrospinal fluid of head-injured patients^9^,^10^. It is known that astrocytes and microglial cells can express complement regulators such as complement receptor type 1-related protein y (Crry), membrane cofactor protein (CD46) and decay accelerating factor (CD55) to avoid complement-mediated damage^10^,^11^. However, neurons are selectively susceptible to complement attack, as they express low levels of complement regulators^10^,^11^. Moreover, these complement regulators which are found in healthy controls, are virtually absent in ischaemic brains^10^,^11^. Therefore, the activation of the complement system must be tightly modulated. Some researchers have reported that blockade of the C3a receptor (C3aR) and C5a receptor (C5aR) in a mouse model of intracerebral haemorrhage markedly reduced neurological impairment, brain water content and granulocyte infiltration^5^,^12^. Furthermore, in animal closed head injury (CHI) models, overexpression of Crry, a C3 convertase inhibitor, improved neurological outcomes and BBB function^13^. In addition, systemic injection of the recombinant Crry molecule in a mouse model of CHI resulted in significant neurological improvement and up-regulation of CD59, a negative regulator of C5b-9 in the injured hemisphere^14^.

In recent years, advances in stem-cell-based therapies have offered great potential to treat neurological deficits^15^. For example, embryonic stem cell (ESC), induced pluripotent stem cell (iPSC) and neural stem cell (NSC) grafts can exert beneficial effects on the restoration of cognition via cell replacement, trophic support and/or immune modulation after brain injury^15^,^16^. Moreover, we previously reported that intracerebral transplantation of induced neural stem cells (iNSCs) generated directly from autologous somatic cells can promote neurological functional recovery in middle cerebral artery occlusion and CHI animals^17^,^18^. Remarkably, we discovered that local iNSC grafts have the potential to modulate CNS inflammation following trauma. Interestingly, several studies have demonstrated that stem cells express complement receptors (CR2, C3aR and C5aR) and regulators (Crry, CD46, CD55 and CD59), suggesting an interaction between stem cells and the complement system^10^,^19^,^20^. In addition, many researchers have found that the systemic delivery of stem cells is a less invasive and therapeutically effective form of administration for treating inflammatory disorders^21^,^22^. However, the effects of systemically injected iNSCs on complement activation after CNS insult remains uncertain.

In the present study, we aimed to explore the role of systemically delivered iNSCs in complement activation following CNS injury. Our data showed that iNSC grafts decreased the levels of sera C3a and C5a and down-regulated the expression of C3d, C9, active Caspase-3 and Bax in the brain, kidney and lung tissues of CHI mice. Furthermore, iNSC grafts decreased the levels of C3d^+^/NeuN^+^, C5b-9^+^/NeuN^+^, C3d^+^/Map2^+^ and C5b-9^+^/Map2^+^ neurons in the injured cortices of CHI mice. Subsequently, we explored the mechanisms underlying these effects. With flow cytometry analysis, we observed a dramatic increase in Crry expression in iNSCs after CHI mouse serum treatment. Moreover, both *in vitro* and *in vivo* loss-of-function studies revealed that iNSCs could modulate complement activation via Crry expression.

## Results

### INSC grafts decreased the levels of sera C3a and C5a and down-regulated the expression of C3d, C9, active Caspase-3 and Bax in the brain, kidney and lung tissues of CHI mice

**Figure 1:**
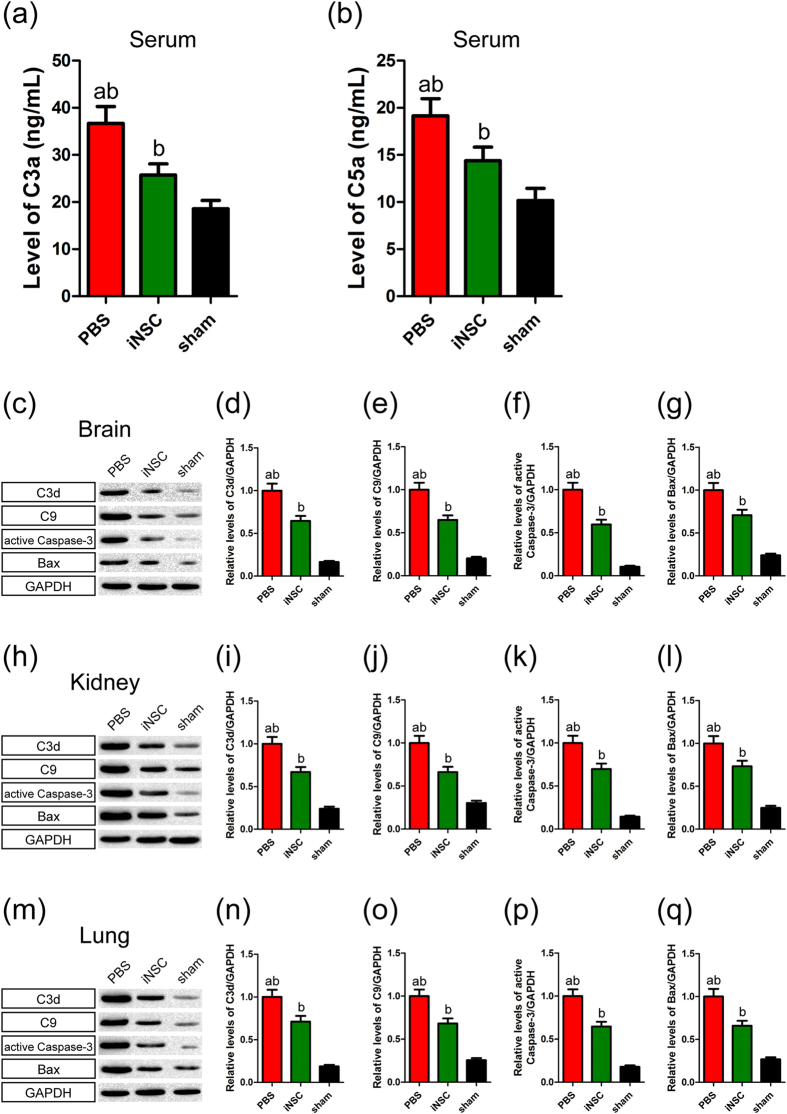
INSC grafts decreased the levels of sera C3a and C5a and down-regulated the expression of C3d, C9, active Caspase-3 and Bax in the brain, kidney and lung tissues of CHI mice. (**a**,**b**) Histograms showed the levels of sera C3a (**a**) and C5a (**b**) among the PBS, iNSC and sham groups on day 7 post-CHI (n = 6/group; (**a**) *P* < 0.05 versus iNSC group; (**b**) *P* < 0.05 versus sham group). (**c**) Representative immunoblots depicted the levels of C3d, C9, active Caspase-3 and Bax in the brain among the three groups on day 7 post-CHI. (**d**–**g**) Histograms showed the relative levels of C3d (**d**), C9 (**e**), active Caspase-3 (**f**), and Bax (**g**) in the brain among the three groups on day 7 post-CHI (n = 6/group; (**a**) *P* < 0.05 versus iNSC group; (**b**) *P* < 0.05 versus sham group). (**h**) Representative immunoblots depicted the levels of C3d, C9, active Caspase-3 and Bax in the kidney among the three groups on day 7 post-CHI. (**i**–**l**) Histograms showed the relative levels of C3d (**i**), C9 (**j**), active Caspase-3 (**k**), and Bax (**l**) in the kidney among the three groups on day 7 post-CHI (n = 6/group; (**a**) *P* < 0.05 versus iNSC group; (**b**) *P* < 0.05 versus sham group). (**m**) Representative immunoblots depicted the levels of C3d, C9, active Caspase-3 and Bax in the lung among the three groups on day 7 post-CHI. (**n**–**q**) Histograms showed the relative levels of C3d (**n**), C9 (**o**), active Caspase-3 (**p**), and Bax (**q**) in the lung among the three groups on day 7 post-CHI (n = 6/group; (**a**) *P* < 0.05 versus iNSC group; (**b**) *P* < 0.05 versus sham group).

CHI models were established using a standardized weight-drop device^23^. At 12 h after CHI, mice received an intravenous injection of 5 × 10^6^ cells. On day 7 post-CHI, GFP-expressing iNSCs were detected in the brain, spleen, kidney, liver and lung (Supplementary Table S1). To study the effect of iNSC grafts on complement activation after CNS trauma, we evaluated serum concentrations of C3a and C5a (Fig. 1a,b). Serum C3a and C5a levels were significantly higher in the CHI group than in the sham group (n = 6/group, *P* < 0.05). Moreover, in contrast to PBS treatment, iNSC administration attenuated CHI-induced increases in serum C3a and C5a levels (*P* < 0.05).

Next, we carried out western blot analysis to determine the protein expression of C3d, C9, active Caspase-3 and Bax in the brain, kidney and lung on day 7 after CHI (Fig. 1c–q). Basal levels of C3d, C9, active Caspase-3 and Bax were low in these tissues of the sham-injured mice. However, CHI induced significant increases in these protein expression in the brain, kidney and lung (n = 6/group, *P* < 0.05). In addition, the expression of C3d, C9, active Caspase-3 and Bax in these tissues was markedly lower in the iNSC group than in the PBS group (*P* < 0.05). Our data suggested that the level of complement activation was positively correlated with the severity of brain, kidney and lung injury post-CHI. Moreover, systemic administration of iNSCs had the potential to attenuate the activation of the complement system and reduce the negative effects of complement activation on secondary damage following CHI.

### INSC grafts decreased the levels of C3d^+^/NeuN^+^, C5b-9^+^/NeuN^+^, C3d^+^/Map2^+^ and C5b-9^+^/Map2^+^ neurons in the injured cortices of CHI mice

**Figure 2:**
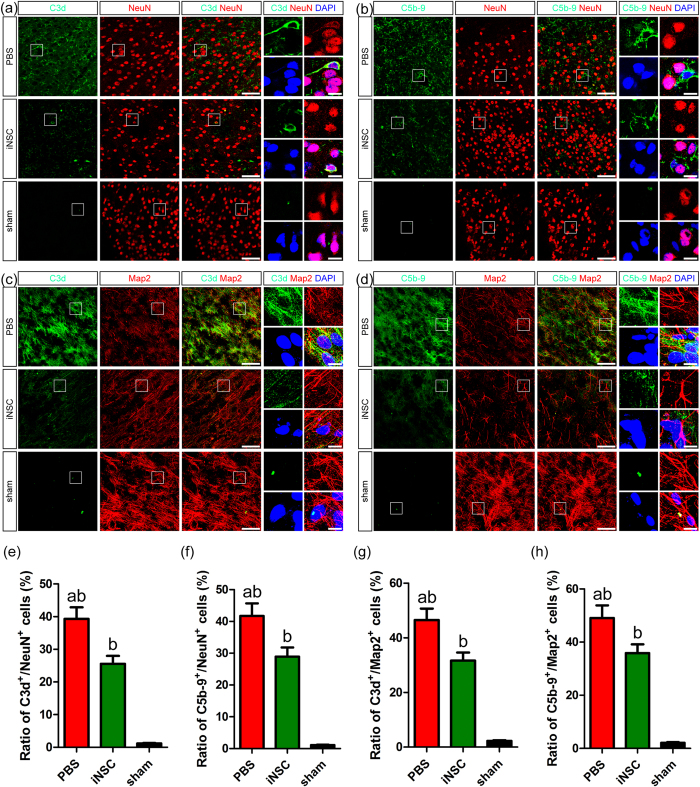
INSC grafts decreased the levels of C3d^+^/NeuN^+^, C5b-9^+^/NeuN^+^, C3d^+^/Map2^+^ and C5b-9^+^/Map2^+^ neurons in the injured cortices of CHI mice. (**a**,**b**) Representative staining for C3d^+^ (green, **a**), C5b-9^+^ (green, **b**), and NeuN^+^ (red) cells depicted the distribution of C3d^+^/NeuN^+^ (**a**) and C5b-9^+^/NeuN^+^ (**b**) neurons in the injured cortex among the PBS, iNSC and sham groups on day 7 post-CHI. Nuclei were counterstained with DAPI (blue). (**c**,**d**) Representative staining for C3d^+^ (green, **c**), C5b-9^+^ (green, **d**), and Map2^+^ (red) cells depicted the distribution of C3d^+^/Map2^+^ (**c**) and C5b-9^+^/Map2^+^ (**d**) neurons in the injured cortex among the three groups on day 7 post-CHI. Nuclei were counterstained with DAPI (blue). (**e**–**h**) Histograms showed the numbers of C3d^+^/NeuN^+^ (**e**), C5b-9^+^/NeuN^+^ (**f**), C3d^+^/Map2^+^ (**g**), and C5b-9^+^/Map2^+^ (**h**) cells in the injured cortex among the three groups on day 7 post-CHI (n = 6/group; (**a**) *P* < 0.05 versus iNSC group; (**b**) *P* < 0.05 versus sham group). Scale bar = 25 μm (5 μm).

To determine the role of iNSC transplantation in neuronal survival following complement activation, we performed double-labelling experiments to measure the levels of C3d^+^/NeuN^+^, C5b-9^+^/NeuN^+^, C3d^+^/Map2^+^ and C5b-9^+^/Map2^+^ neurons in the injured cortex on day 7 post-CHI (Fig. 2). An average of 20 equally spaced slides (100-μm intervals) containing samples from throughout the injured cortex (a series of 5-mm coronal sections) of each brain was assessed^18^. Cortical fields (0.73 mm^2^ each) in both the lesion core and the peri-lesion area were selected from each brain, and the numbers of positively stained cells in these fields were counted^18^. All of these double-labelling cells were rarely detected in the sham group. However, staining of brain cryosections from CHI mice showed that C3d and C5b-9 immunostaining was detected in the NeuN^+^ and Map2^+^ neurons in the injured cortex. Furthermore, the levels of C3d^+^/NeuN^+^, C5b-9^+^/NeuN^+^, C3d^+^/Map2^+^ and C5b-9^+^/Map2^+^ neurons were significantly lower in the iNSC group than in the PBS group (n = 6/group, *P* < 0.05). These findings revealed that systemic administration of iNSCs could efficiently decrease the detrimental effects of complement activation on neurons after CHI.

### Expression of complement regulators in stem cells

**Figure 3:**
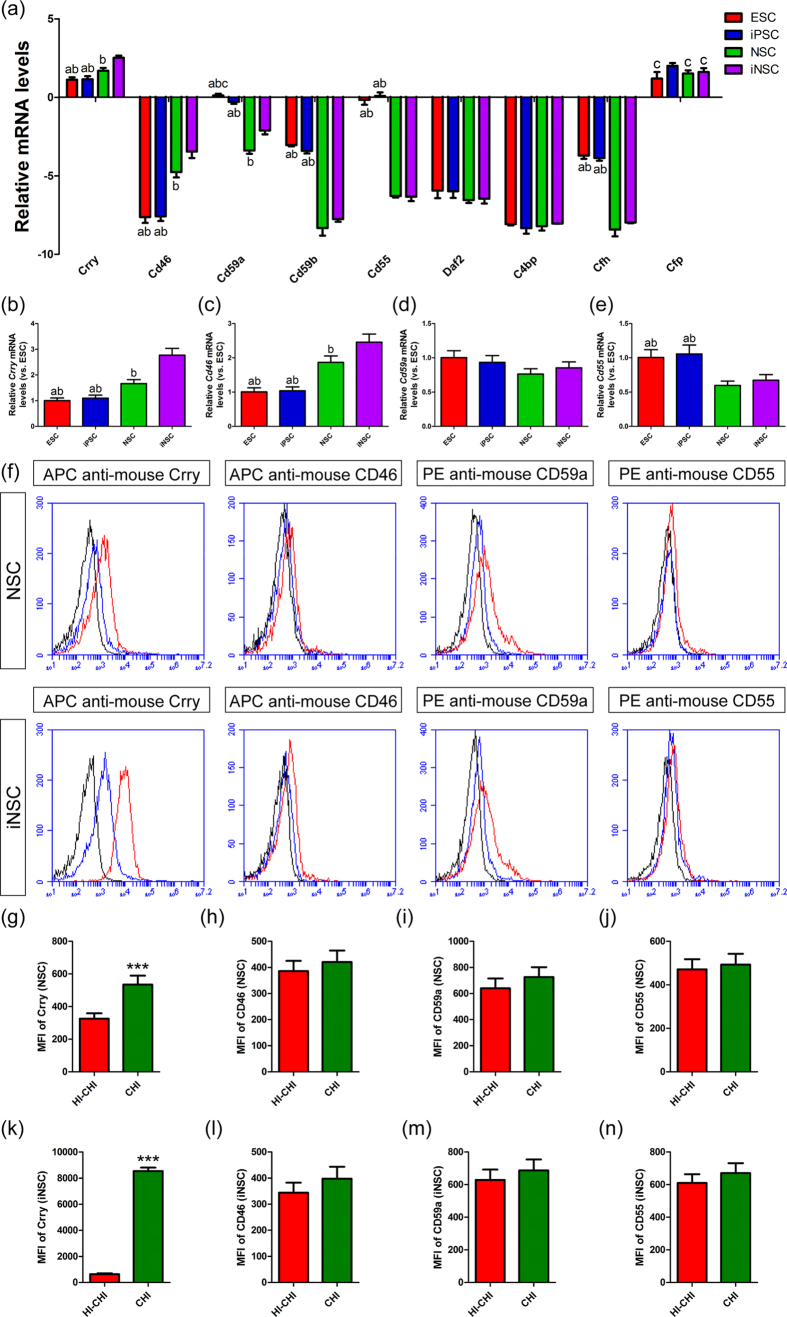
Expression of complement regulators in stem cells. (**a**) The levels of *Crry, Cd46, Cd59a, Cd59b, Cd55, Daf2, C4bp, Cfh* and *Cfp* genes in ESCs, iPSCs, NSCs and iNSCs were determined using Agilent mRNA expression arrays and analysed after signal values normalization (n = 3/group; (**a**) *P* < 0.05 versus NSCs; (**b**) *P* < 0.05 versus iNSCs; (**c**) *P* < 0.05 versus iPSCs). (**b–e**) The expression of *Crry* (**b**), *Cd46* (**c**), *Cd59a* (**d**), and *Cd55* (**e**) genes in ESCs, iPSCs, NSCs and iNSCs were determined by RT-QPCR (n = 3/group; (**a**) *P* < 0.05 versus NSCs; (**b**) *P* < 0.05 versus iNSCs). (**f**) Representative flow cytometric analysis of Crry, Cd46, Cd59a and Cd55 expression in NSCs and iNSCs after CHI (red) or heat-inactivated CHI (HI-CHI, blue) mouse serum administration. Isotype antibodies were used as controls (black). (**g–n**) Histograms showed the median fluorescence intensity (MFI) values of Crry, Cd46, Cd59a and Cd55 expression in NSCs (**g–j**) and iNSCs (**k–n**) (n = 6/group; ***P < 0.001).

To explore the mechanism underlying the effects of the iNSC grafts described above, we examined the expression of complement receptors and regulators among syngeneic mouse ESCs, iPSCs, NSCs and iNSCs. Based on the global gene expression profiles of these stem cells, which were determined using Agilent mRNA expression arrays, we found that *Cd59a, Cd59b, Cd55* and *Cfh* levels in NSCs and iNSCs were lower than those in ESCs and iPSCs (n = 3/group, *P* < 0.05) (Fig. 3a). In contrast, *Crry* and *Cd46* levels in NSCs and iNSCs were higher than those in ESCs and iPSCs (*P* < 0.05). Furthermore, *Crry, Cd46* and *Cd59a* levels in iNSCs were higher than those in NSCs (*P* < 0.05).

To verify the microarray results, we analysed the expression of *Crry, Cd46, Cd59a* and *Cd55* in ESCs, iPSCs, NSCs and iNSCs with RT-QPCR (Fig. 3b–e). The relative levels of *Crry* and *Cd46* genes in NSCs and iNSCs were substantially higher than those in ESCs and iPSCs (n = 3/group, *P* < 0.05). Additionally, *Crry* and *Cd46* levels in iNSCs were higher than those in NSCs (*P* < 0.05). However, *Cd59a* levels were not significantly different in these stem cells. In addition, *Cd55* levels in ESCs and iPSCs were markedly higher than those in NSCs and iNSCs (*P* < 0.05).

To identify the regulatory effects of stem cells on complement activation, we utilized flow cytometry to determine Crry, Cd46, Cd59a and Cd55 protein expression levels in NSCs and iNSCs after CHI or heat-inactivated CHI (HI-CHI) mouse serum treatment (Fig. 3f–n). Flow cytometry analysis revealed a dramatic increase in Crry expression in NSCs and iNSCs in the CHI group compared with the HI-CHI group (n = 6/group, *P* < 0.05). In contrast, Cd46, Cd59a and Cd55 expression in NSCs and iNSCs exhibited no significant differences between the CHI and HI-CHI groups. In short, our results implied that Crry, an important complement regulator, had the potential to mediate the communication between iNSCs and the complement system.

### INSCs modulated complement activation via Crry expression

**Figure 4:**
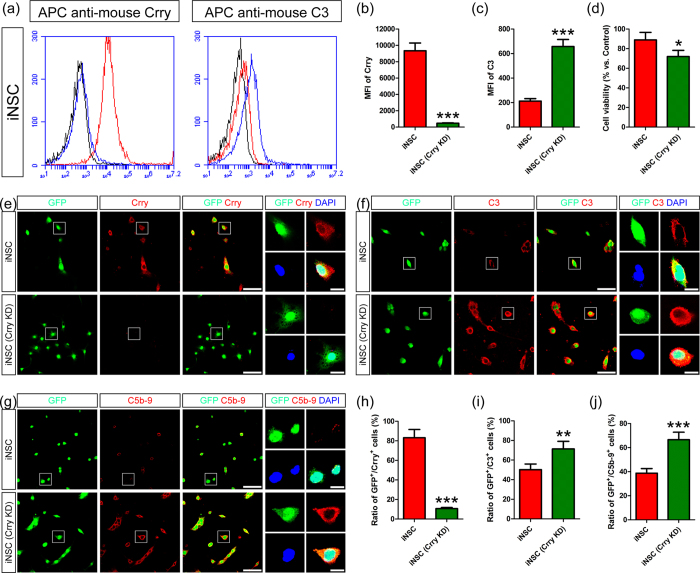
INSCs modulated complement activation via Crry expression. (**a**) Representative flow cytometric analysis of Crry and C3 expression in iNSCs (red) and iNSCs (Crry KD, blue) after CHI mouse serum administration. Isotype antibodies were used as controls (black). (**b**,**c**) Histograms showed the MFI values of Crry and C3 expression in iNSCs and iNSCs (Crry KD) (n = 6/group; ***P < 0.001). (**d**) INSCs and iNSCs (Crry KD) were treated with CHI mouse serum for 45 min. Then, cell viability was detected by MTT assay. The viability of iNSCs without CHI mouse serum treatment was considered to be at 100% (n = 3/group; *P < 0.05). (**e–g**) Representative staining for Crry^+^ (red, **e**), C3^+^ (red, **f**), and C5b-9^+^ (red, **g**) depicted Crry, C3 and C5b-9 expression levels in GFP-labelled iNSCs (green) and iNSCs (Crry KD) (green) after CHI mouse serum administration. Nuclei were counterstained with DAPI (blue). (**h–j**) Histograms indicated the numbers of GFP^+^/Crry^+^ (**h**), GFP^+^/C3^+^ (**i**), and GFP^+^/C5b-9^+^ (**j**) cells between the two groups after CHI mouse serum administration (n = 6/group; **P < 0.01; ***P < 0.001). Scale bar = 10 μm (5 μm).

To determine the role of Crry expression in mediating the regulatory effects of iNSCs on complement activation, we carried out a loss-of-function study to knock down (KD) the levels of Crry in iNSCs by Crry-specific shRNA lentiviral particles (Supplementary Figure S1). After CHI mouse serum treatment, Crry expression levels in the iNSC (Crry KD) group were substantially lower than those in the iNSC group (n = 6/group, *P* < 0.05) (Fig. 4a,b). In contrast, C3 expression levels were markedly higher in the iNSC (Crry KD) group than in the iNSC group (*P* < 0.05) (Fig. 4a,c).

Next, we performed the 3-[4, 5-dimethylthiazol-2-yl]-2, 5-diphenyl tetrazolium bromide (MTT) assay to measure cell viability in the iNSC and iNSC (Crry KD) groups. There was no significant difference in the cell viability between the iNSC and iNSC (Crry KD) groups without CHI mouse serum treatment (Supplementary Figure S2a). In addition, the levels of NeuN^+^ neurons, GFAP^+^ astrocytes, and Olig2^+^ oligodendrocytes derived from iNSCs and iNSCs (Crry KD) had no significant differences (Supplementary Figure S2b–d). However, iNSCs (Crry KD) treated with CHI mouse serum for 45 min displayed an obvious reduction in cell viability (Fig. 4d). Moreover, the cellular viability of iNSCs (Crry KD) was markedly lower than that of iNSCs after CHI mouse serum treatment (n = 3/group, *P* < 0.05).

We also carried out immunofluorescence assay by confocal laser scanning microscopy (CLSM) to study the role of Crry expression in iNSCs. First, Crry expression levels in iNSCs and iNSCs (Crry KD) were detected after CHI mouse serum treatment (Fig. 4e). CLSM revealed clear GFP expression in iNSCs and iNSCs (Crry KD). We found that the levels of GFP^+^/Crry^+^ cells in the iNSC group were significantly higher than those in the iNSC (Crry KD) group (n = 6/group, *P* < 0.05) (Fig. 4h). Then, we analysed the deposition of C3 and C5b-9 in iNSCs and iNSCs (Crry KD) after CHI mouse serum treatment (Fig. 4f,g). Quantitatively, the levels of GFP^+^/C3^+^ and GFP^+^/C5b-9^+^ cells were substantially lower in the iNSC group than in the iNSC (Crry KD) group (*P* < 0.05) (Fig. 4i,j). The *in vitro* study suggested that Crry expression levels in iNSCs were negatively correlated with the harmful effects of complement activation. Moreover, iNSCs could attenuate the adverse effects of complement activation via Crry expression.

### INSC grafts modulated complement activation in CHI mice via Crry expression

**Figure 5:**
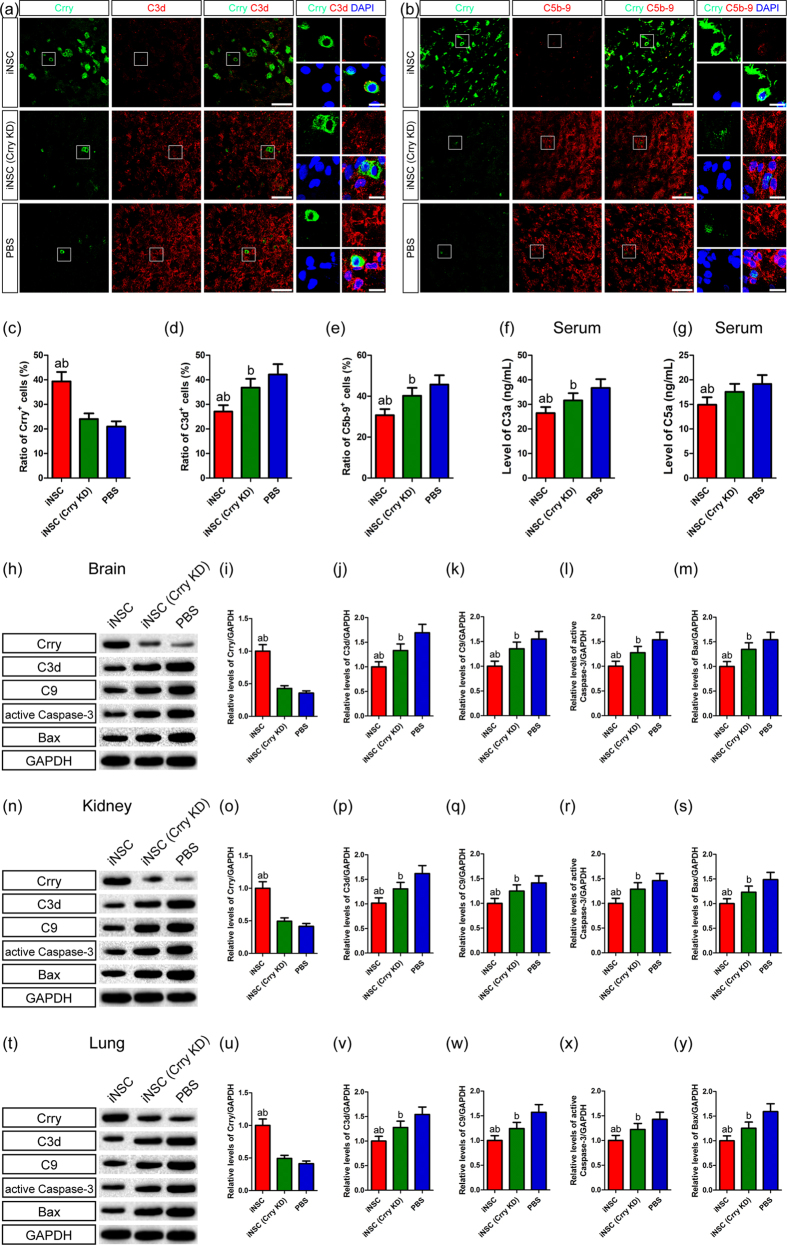
INSC grafts modulated complement activation in CHI mice via Crry expression. (**a**,**b**) Representative staining for C3d^+^ (red, **a**), C5b-9^+^ (red, **b**), and Crry^+^ (green) cells depicted the distribution of C3d^+^/Crry^+^ (**a**) and C5b-9^+^/Crry^+^ (**b**) cells in the injured cortex among the iNSC, iNSC (Crry KD) and PBS groups on day 7 post-CHI. Nuclei were counterstained with DAPI (blue). (**c–e**) Histograms indicated the numbers of Crry^+^ (**c**), C3d^+^ (**d**), and C5b-9^+^ (**e**) cells in the injured cortex among the three groups on day 7 post-CHI (n = 6/group; (**a**) *P* < 0.05 versus iNSC (Crry KD) group; (**b**) *P* < 0.05 versus PBS group). (**f**,**g**) Histograms showed the levels of sera C3a (**f**) and C5a (**g**) among the three groups on day 7 post-CHI (n = 6/group; (**a**) *P* < 0.05 versus iNSC (Crry KD) group; (**b**) *P* < 0.05 versus PBS group). (**h**) Representative immunoblots depicted the levels of Crry, C3d, C9, active Caspase-3 and Bax in the brain among the three groups on day 7 post-CHI. (**i–m**) Histograms showed the relative levels of Crry (**i**), C3d (**j**), C9 (**k**), active Caspase-3 (**l**), and Bax (**m**) in the brain among the three groups on day 7 post-CHI (n = 6/group; (**a**) *P* < 0.05 versus iNSC (Crry KD) group; (**b**) *P* < 0.05 versus PBS group). (**n**) Representative immunoblots depicted the levels of Crry, C3d, C9, active Caspase-3 and Bax in the kidney among the three groups on day 7 post-CHI. (**o-s**) Histograms showed the relative levels of Crry (**o**), C3d (**p**), C9 (**q**), active Caspase-3 (**r**), and Bax (**s**) in the kidney among the three groups on day 7 post-CHI (n = 6/group; (**a**) *P* < 0.05 versus iNSC (Crry KD) group; (**b**) *P* < 0.05 versus PBS group). (**t**) Representative immunoblots depicted the levels of Crry, C3d, C9, active Caspase-3 and Bax in the lung among the three groups on day 7 post-CHI. (**u–y**) Histograms showed the relative levels of Crry (**u**), C3d (**v**), C9 (**w**), active Caspase-3 (**x**), and Bax (**y**) in the lung among the three groups on day 7 post-CHI (n = 6/group; (**a**) *P* < 0.05 versus iNSC (Crry KD) group; (**b**) *P* < 0.05 versus PBS group). Scale bar = 25 μm (5 μm).

To identify the regulatory effects of iNSC grafts on complement activation via Crry expression, we performed double-labelling experiments to measure the deposition of C3d and C5b-9 in the injured cortex on day 7 post-CHI (Fig. 5a,b). We found that the levels of Crry^+^ cells in the iNSC group were markedly higher than those in the iNSC (Crry KD) or PBS groups (n = 6/group, *P* < 0.05) (Fig. 5c). However, the levels of C3d^+^ and C5b-9^+^ cells were significantly lower in the iNSC group than in the iNSC (Crry KD) group (*P* < 0.05) (Fig. 5d,e). Additionally, C3d^+^ and C5b-9^+^ cell levels were markedly lower in the iNSC (Crry KD) group than in the PBS group (*P* < 0.05). Next, we discovered that serum C3a and C5a levels were substantially higher in the iNSC (Crry KD) group than in the iNSC group (n = 6/group, *P* < 0.05) (Fig. 5f,g). Moreover, serum C3a levels were significantly higher in the PBS group than in the iNSC (Crry KD) group (*P* < 0.05).

Furthermore, we carried out western blot analysis to determine the protein expression of Crry, C3d, C9, active Caspase-3 and Bax in the brain, kidney and lung on day 7 after CHI (Fig. 5h–y). Crry expression levels in these tissues of CHI mice that received iNSC transplants were significantly higher than those of CHI mice that received iNSC (Crry KD) transplants or PBS (n = 6/group, *P* < 0.05). In addition, the levels of C3d, C9, active Caspase-3 and Bax in the brain, kidney and lung tissues of the iNSC (Crry KD) group were markedly higher than in the iNSC group but were significantly lower than those in the PBS group (*P* < 0.05). Immunofluorescence assay also revealed that the numbers of C3d^+^, C5b-9^+^, active Caspase-3^+^ and Bax^+^ cells in the brain, kidney and lung tissues of the iNSC (Crry KD) group were substantially higher than in the iNSC group but were markedly lower than those in the PBS group (*P* < 0.05) (Supplementary Figures S3–S5). The *in vivo* study further demonstrated that systemic administration of iNSCs could decrease the detrimental effects of complement activation via Crry expression.

### INSC grafts reduced neurological deficits, cerebral edema, BBB permeability and inflammatory infiltration in CHI mice via Crry expression

**Figure 6:**
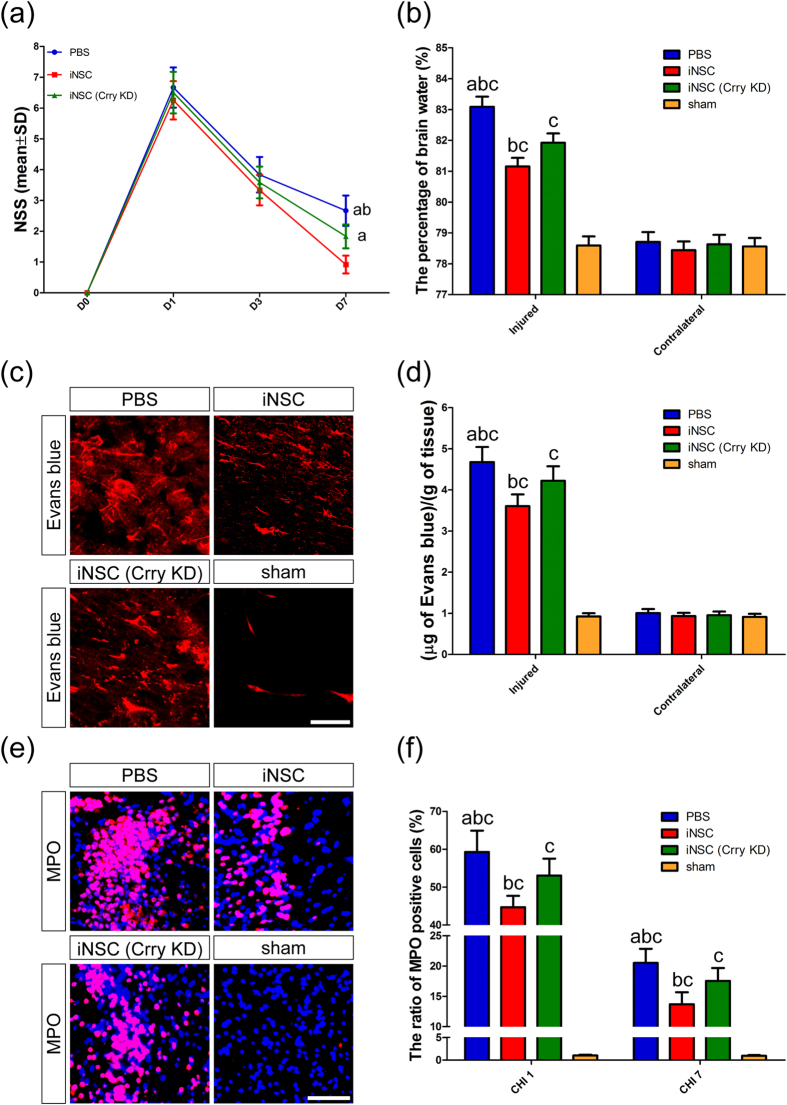
INSC grafts reduced neurological deficits, cerebral edema, BBB permeability and neutrophil infiltration in CHI mice via Crry expression. (**a**) Assessment of neurological impairment post-CHI by NSS among the PBS, iNSC and iNSC (Crry KD) groups from CHI 0 through CHI 7 (n = 12/group; (**a**) *P* < 0.05 versus iNSC group; (**b**) *P* < 0.05 versus iNSC (Crry KD) group). (**b**) Cerebral edema analysis indicated the percentages of brain water in the injured and contralateral hemispheres among the four groups on day 7 post-CHI (n = 6/group; (**a**) *P* < 0.05 versus iNSC group; (**b**) *P* < 0.05 versus iNSC (Crry KD) group; (**c**) *P* < 0.05 versus sham group). (**c**) Representative Evans blue fluorescence in the injured hemispheres of mice among the four groups on day 7 post-CHI. (**d**) BBB permeability analysis indicated the concentrations of Evans blue in the injured and contralateral hemispheres among the four groups on day 7 post-CHI (n = 6/group; (**a**) *P* < 0.05 versus iNSC group; (**b**) *P* < 0.05 versus iNSC (Crry KD) group; (**c**) *P* < 0.05 versus sham group). (**e**) Representative staining for MPO^+^ (red) cells depicted the distribution of MPO^+^ neutrophils in the injured cortex among the four groups on day 1 post-CHI. (**f**) Histograms showed the numbers of MPO^+^ cells in the injured cortex among the four groups on day 1 and 7 post-CHI (n = 6/group; (**a**) *P* < 0.05 versus iNSC group; (**b**) *P* < 0.05 versus iNSC (Crry KD) group; (**c**) *P* < 0.05 versus sham group). Scale bar = 200 μm (**c**); 100 μm (**e**).

The neurological function of CHI mice was observed to determine the therapeutic efficacy of iNSC grafts. Neurological impairment post-CHI was assessed with the neurological severity score (NSS) (n = 12/group) (Fig. 6a). Healthy mice with an NSS of 0 were enrolled in the study. Two-way ANOVA indicated that time (*F* = 695.742, *P* < 0.001), treatment (*F* = 24.806, *P* < 0.001), and the interaction between time and treatment (*F* = 5.839, *P* < 0.001) exhibited significant effects in terms of NSS. Simple main effect analysis indicated that NSS was markedly different among the PBS, iNSC and iNSC (Crry KD) groups at 7 days (*F* = 32.032, *P* < 0.001), but not at 1 (*F* = 1.839, *P* = 0.164) or 3 days (*F* = 2.613, *P* = 0.078) after CHI. On day 7 post-CHI, NSS was greater in the PBS group (2.67 ± 0.49, *P* < 0.05) than in the iNSC (0.92 ± 0.29) or iNSC (Crry KD) groups (1.83 ± 0.39) and was lower in the iNSC group than in the iNSC (Crry KD) group. The simple main effect of time was significant in the PBS (*F* = 176.903, *P* < 0.001), iNSC (*F* = 298.161, *P* < 0.001), and iNSC (Crry KD) groups (*F* = 232.355, *P* < 0.001). Post hoc Tukey’s tests (at *P* ≤ 0.05) were utilized to identify these effects. The simple main effect of time was significantly different in the PBS, iNSC and iNSC (Crry KD) groups among 1, 3 and 7 days after CHI with respect to NSS. The NSS was lower at 3 days than at 1 day post-CHI and was higher at 3 days than at 7 days post-CHI in the PBS, iNSC and iNSC (Crry KD) groups. Sham animals did not show any signs of neurological impairment (data not shown).

Cerebral edema and BBB permeability analyses were performed on day 7 post-CHI to further evaluate the beneficial effects of iNSC administration (Fig. 6b–d). CHI induced significant increases in cerebral edema and BBB permeability in the injured hemispheres of CHI mice compared to sham mice (n = 6/group, *P* < 0.05). It revealed that the percentages of brain water and the concentrations of Evans blue in the injured hemispheres of the iNSC (Crry KD) group were markedly higher than those in the injured hemispheres of the iNSC group but were significantly lower than those in the PBS group (*P* < 0.05). However, there were no significant intergroup differences in terms of brain water content and Evans blue leakage in the contralateral hemispheres.

HE staining showed that resident cells were well arranged in the cerebral hemispheres of sham-injured mice. In contrast, the structure of the injured brain hemispheres of CHI mice was broken, accompanied by tissue necrosis and immune cell infiltration (data not shown). To measure the degree of inflammatory infiltrate, we detected the levels of neutrophils (MPO), astrocytes (GFAP), and microglia/macrophages (Iba1) in the brain and discovered that the numbers of MPO^+^, GFAP^+^ and Iba1^+^ cells were increased on day 1 and 7 post-CHI (n = 6/time point/group, *P* < 0.05) (Fig. 6e,f and Supplementary Figure S6). However, iNSC transplantation partially attenuated neutrophil recruitment, reactive astrogliosis and microglia/macrophage infiltration, as noted in PBS-treated mice. In addition, at 7 days after CHI, the levels of MPO^+^ neutrophils, GFAP^+^ astrocytes and Iba1^+^ microglia/macrophages in the injured cortices of the iNSC (Crry KD) group were markedly higher than in the iNSC group but was significantly lower than the levels in the PBS group (*P* < 0.05). Taken together, our findings showed that systemic administration of iNSCs could reduce neurological deficits, cerebral edema, BBB permeability and inflammatory infiltration in CHI mice via Crry expression.

## Discussion

The complement system has important roles in host defence, debris removal and immune modulation, but we should be aware that it can become detrimental when excessively activated^5^. In this study, we found that CHI-induced complement activation marked by significant increases in serum C3a and C5a levels was associated with neurological impairment, cerebral edema, BBB dysfunction and inflammatory infiltration. Further, the protein expression of C3d, C9, active Caspase-3 and Bax in the brain, kidney and lung were dramatically elevated after CHI, suggesting that the complement cascade was involved in cell apoptosis in multiple organs. Moreover, prominent deposition of C3d and C5b-9 in the NeuN^+^ and Map2^+^ neurons in the injured cortices of CHI mice also revealed that complement activation could contribute to neuronal injury. However, these detrimental effects were attenuated by systemic administration of iNSCs. Additionally, the levels of sera C3a and C5a; the protein expression of C3d, C9, active Caspase-3 and Bax in the brain, kidney and lung; and the numbers of C3d^+^ and C5b-9^+^ neurons were significantly reduced by iNSC grafts.

We subsequently explored the mechanisms underlying these therapeutic effects of iNSC transplants. Analyses of global gene expression profiles and RT-QPCR indicated that the levels of *Crry* and *Cd46* genes in NSCs were markedly higher than those in ESCs and iPSCs but were substantially lower than those in iNSCs. Using HI-CHI mouse serum as a negative control, the flow cytometry assay showed a dramatic increase in Crry expression in iNSCs treated with CHI mouse serum, which was a source of complement^24^. As mentioned previously, Crry, a rodent complement regulator that is similar in function to human CD46 and CD55, can inhibit C3 convertase, which modulates complement activation and reduces downstream products, including anaphylatoxins and C5b-9^13^,^14^. Thus, we assume that iNSCs may regulate the activation of the complement cascade via enhanced expression of complement regulators, especially Crry. Subsequently, we performed loss-of-function studies both *in vitro* and *in vivo* to clarify the role of Crry expression in iNSCs.

Our data indicated that the down-regulation of Crry in iNSCs had no remarkable influence on their survival, proliferation and differentiation *in vitro*. However, following CHI mouse serum treatment, iNSCs (Crry KD) displayed a significant reduction in cell viability. In addition, the absence of Crry was associated with extensive deposits of C3 and C5b-9 on the membrane of iNSCs (Crry KD) that were treated with CHI mouse serum. These findings suggest that suppression of Crry in iNSCs may increase the risk of complement-mediated damage, which is in agreement with previous studies^25^,^26^,^27^. An *in vivo* loss-of-function study also revealed that systemic delivery of iNSCs (Crry KD) failed to increase the levels of Crry in the brain, kidney and lung. Moreover, Crry deficiency was involved in C3d and C5b-9 deposition in the injured cortices of CHI mice. Additionally, the lack of Crry led to elevated levels of C3d, C9, active Caspase-3 and Bax in multiple organs of CHI mice following iNSC (Crry KD) administration. These results indicate that iNSC grafts modulate complement activation post-CHI mainly through expression of Crry.

The discrepancies between iNSC and iNSC (Crry KD) transplants may be attributable to the role of Crry and/or the viability of cell grafts because iNSCs (Crry KD) are susceptible to complement attack. For instance, we discovered that GFP-expressing cells distributed in the injured hemispheres of CHI mice were reduced in the iNSC (Crry KD) group compared to the iNSC group (data not shown). Hence, if engrafted iNSCs (Crry KD) were damaged by complement-mediated cytolysis, their therapeutic effects on neurological impairment, cerebral edema, BBB dysfunction and inflammatory infiltrate would be disrupted. Certainly, Crry deficiency failed to modulate the activation of the complement system, which would also contribute to these disorders. Although Crry is widely expressed *in vivo*, our experiment, which is consistent with previous findings, suggests that the levels of Crry are relatively insufficient for the development of complement activation post-CHI^10^,^25^,^26^. Therefore, systemically transplanted iNSCs, which have the potential to increase the expression of Crry in response to immune stimuli, can escape from complement-induced damage and regulate complement activation to promote neurological functional recovery in CHI mice.

## Methods

### Closed head injury models

All experimental procedures were in compliance with the Guide for the Care and Use of Laboratory Animals published by the National Institutes of Health (NIH) and approved by the Committee on the Ethics of Animal Experiments of the General Hospital of Beijing Military Region, P.L.A (Permit Number: 2014-044). CHI models were established using a standardized weight-drop device as previously reported^23^. Detailed methods were provided in Supplementary Methods S1. Animals were evaluated at 1 h post-CHI using an NSS by two blinded, trained investigators^23^. Mice having an NSS of 4–8 were randomly assigned to two groups: the iNSC group (mice receiving iNSC transplantation) and the PBS group (mice receiving PBS treatment).

### Cell preparation and transplantation

B6 mouse GFP-expressing ESCs, iPSCs, iNSCs and NSCs were generated and cultured as described previously^17^. Detailed methods were provided in Supplementary Methods S2. For transplantation, cultured cells were digested with accutase (Invitrogen, Carlsbad, CA, USA) and washed three times with PBS. After the density of the single-cell suspension was adjusted, the cells were maintained on ice. At 12 h after CHI, mice were anaesthetized again and randomly selected to receive 200 μl of cell suspension containing 5 × 10^6^ cells or PBS intravenously.

### Evaluation of neurological function, cerebral edema and BBB permeability

CHI-induced neurological impairment was assessed using the NSS. Detailed methods were provided in Supplementary Table S2. In addition, animals were sacrificed after anaesthesia and their fresh or perfused-fixed brain tissues were collected. Cerebral edema and BBB permeability were measured by two blinded, trained investigators as previously described^23^. For analysis of serum complement content, mouse blood harvested via cardiac puncture immediately prior to perfusion was transferred to sterile BD Vacutainer SST^TM^ tubes (BD Biosciences, San Jose, CA, USA) and centrifuged at 3,000 rpm at 4 °C (20 min). The supernatants were collected and stored at −80 °C.

### Morphological analysis

The experiments of hematoxylin and eosin (HE) staining and immunofluorescence were performed as previously described^18^,^28^. Detailed methods were provided in Supplementary Methods S3.

### Agilent mRNA expression array and RT-QPCR assay

The Agilent mRNA expression array was performed by the CapitalBio Company (Beijing, China)^28^. For verification, RNA extracted from cultured cells was assessed by RT-QPCR assay. The sequences of the PCR primer pairs used in this study were reported previously^29^,^30^,^31^,^32^.

### ELISA

The levels of sera C3a (BD Biosciences) and C5a (Abcam, Cambridge, MA, USA) were detected using ELISA kits according to the manufacturer’s protocol.

### Western blot and flow cytometry

The experiments of western blot and flow cytometry were performed as previously described^18^,^28^. Detailed methods were provided in Supplementary Methods S4 and S5.

### Loss-of-function study

To provide evidence of the role of Crry in iNSC administration, we utilized Crry-specific shRNA lentiviral particles (sc-42795-V, Santa Cruz Biotechnology, Santa Cruz, CA, USA) and negative control shRNA lentiviral particles (sc-108080, Santa Cruz Biotechnology) to transfect iNSCs according to the manufacturer’s instructions. GFP-expressing iNSCs were derived from rtTA/GFP transgenic C57BL/6 mouse embryos. In order to acquire iNSCs (Crry KD), puromycin (2–8 μg/ml, Santa Cruz Biotechnology) was used for selection of stably transfected iNSCs (Crry KD). After transfection, Crry expression was assayed by RT-QPCR and western blot.

### Complement deposition assay

CHI mouse serum was collected at 12 h post-trauma as described above. Heat inactivated CHI (HI-CHI) mouse serum, a complement deficient control, was processed by heating to 56 °C for 45 min^24^. For complement deposition assay, cultured cells were digested with accutase, washed with PBS, resuspended in 250 μl of CHI or HI-CHI mouse serum, and seeded into a 24-well plate (1 × 10^5^ cells per well) for 45 min at 37 °C. Afterwards, the cells were washed thoroughly and fixed in 4% PFA in 0.1 M PBS (PH 7.4) for flow cytometry and immunofluorescence.

### Cell viability assay

At the indicated time point following CHI mouse serum treatment, cells were washed thoroughly, seeded in a 96-well plate, and cultured with the iNSC culture medium. Cell viability was measured using an MTT (Sigma-Aldrich, St. Louis, MO, USA) assay according to the manufacturer’s protocol. The net absorbance from the plate of cells without CHI mouse serum treatment was considered to be at 100% cell viability.

### Statistical analysis

The SPSS17.0 statistical software package was used for statistical analysis. Data were presented as mean ± standard deviation (SD). Student’s t-test, One-way ANOVA, two-way ANOVA and simple main effect analyses were conducted to determine statistical significance. A *P* < 0.05 was considered to be significant.

## Additional Information

**How to cite this article**: Gao, M. *et al*. Systemic Administration of Induced Neural Stem Cells Regulates Complement Activation in Mouse Closed Head Injury Models. *Sci. Rep.* **7**, 45989; doi: 10.1038/srep45989 (2017).

**Publisher's note:** Springer Nature remains neutral with regard to jurisdictional claims in published maps and institutional affiliations.

## Supplementary Material

Supplementary Information
