# Supplementary material for: Systemic Administration of Induced Neural Stem Cells Regulates Complement Activation in Mouse Closed Head Injury Models
Source: Sci Rep. 2017 Apr 6;7:45989. doi: 10.1038/srep45989 (PMC5382667; doi:10.1038/srep45989)
Supplement: Supplementary Information [file srep45989-s1.pdf]

Supplementary material for: Systemic Administration of Induced Neural Stem Cells Regulates Complement Activation in Mouse Closed Head Injury Models

Mou Gao [1, 2], Qin Dong [3], Hui Yao [2], Yingzhou Lu [4], Xinchao Ji [2], Mingming Zou [2], Zhijun Yang [2], Minhui Xu [1,*], Ruxiang Xu [2, *]

[1]Department of Neurosurgery, The Third Affiliated Hospital of The Third Military Medical University, Chongqing 400042, China

[2]Affiliated Bayi Brain Hospital, P.L.A Army General Hospital, Beijing 100700, China

[3]Department of Neurology, Fu Xing Hospital, Capital Medical University, Beijing 100038, China

[4]Department of Obstetrics, Fu Xing Hospital, Capital Medical University, Beijing 100038, China

Supplementary Methods S1: Closed head injury models

Healthy adult male C57BL/6 (B6) mice weighing 24~30 g (Vital River Laboratories, Beijing, China) were housed in a temperature- and humidity-controlled room with food and water *ad libitum*. All experimental procedures were in compliance with the Guide for the Care and Use of Laboratory Animals published by the National Institutes of Health (NIH) and approved by the Committee on the Ethics of Animal Experiments of the General Hospital of Beijing Military Region, P.L.A (Permit Number: 2014–044).

Animals were anesthetized with inhalative isoflurane (induction: 3% isoflurane; maintenance: 1.25% isoflurane) and received fentanyl (0.05 mg/kg body weight per day, intraperitoneal injection) as the analgesic agent. The parietal bone was exposed by a midline scalp incision after shaving and cleaning the skin. A free-falling rod with a blunt tip of 3.0 mm diameter was dropped onto the mouse's skull (2.0 mm anterior to the lambda suture and 2.0 mm lateral to the middle line) at a falling height of 3.0 cm. Then the scalp wound was sutured and treated with povidone-iodine solution. After surgery, mice were allowed to recover on a heating pad until fully awake. Sham-operated mice underwent the same procedures (anesthesia, analgesia, and scalp incision), but not head trauma.

Supplementary Methods S2: INSC generation

Briefly, GFP-expressing mouse embryonic fibroblasts (MEF) were cultured in Dulbecco's modified Eagle's medium (DMEM) (Invitrogen, Carlsbad, CA, USA) containing 10% fetal bovine serum (FBS) (Invitrogen) and 0.1mM non-essential amino acids (NEAA) (Invitrogen), infected with combinations of lentiviruses (TetO-FUW-Oct4, Sox2, Klf4 and c-Myc), and

subsequently treated with doxycycline (DOX, 2-8 μg/ml) (Sigma-Aldrich, St. Louis, MO, USA) for 6 days. In the final stage of reprogramming, the medium was changed to iNSC culture medium (Neurobasal: DMEM/F12 (1:1) containing 2% B27 supplements, 20 ng/ml basic fibroblast growth factor (bFGF), 20 ng/ml epidermal growth factor (EGF), 0.05% bovine serum albumin (BSA) and 2 mM L-glutamine) (all from Invitrogen). INSC clones appeared in 13-26 days after induction and were expanded.

Supplementary Methods S3: Morphological analysis

Slides of tissues and cultured cells were blocked for 1 h using 10% BSA/0.3% TritonX-100 and then incubated overnight at 4°C with primary antibodies (Supplementary Table S3). After being washed in PBS, they were incubated for 1~2 h at room temperature (RT) with secondary antibodies (Supplementary Table S3). After several washes with PBS, the nuclei were stained with Dapi Fluoromount-G (SouthernBiotech, Birmingham, AL, USA) and staining was detected via fluorescent microscopy (DM3000, Leica) or CLSM (TCS SP5 II, Leica). The number of positive cells was manually counted directly on the microscopy at 20 x magnification and adjusted using image analysis software (Image-Pro plus 5.0). The ratio of positive cells was calculated as (number of positive cells/total number of cells) x100%.

Supplementary Methods S4: Western blot

Protein concentrations were determined using the BCA assay (Thermo Scientific, Hudson, NH, USA). Protein samples were heated for 10 min at 95°C and separated by SDS-PAGE (35 μg per lane), and then transferred to PVDF membranes (Millipore, Bedford, MA, USA). The

blots were blocked for 1 h at RT with 5% BSA in TBST and then detected by incubation with primary antibodies (Supplementary Table S3) at 4°C overnight. After several washes, the blots were incubated for 1 h at RT with HRP-conjugated secondary antibodies (Supplementary Table S3). Immunoblots were visualized using the SuperSignal ECL (Pierce, Rockford, IL, USA). The results expressed relative to the control and normalized to GAPDH.

Supplementary Methods S5: Flow cytometry

After digestion, the Fc receptors of cultured cells were blocked with TruStain fcXTM reagent (Biolegend, San Diego, CA, USA) for 10 min on ice. Then cells were incubated with cell surface antibodies (Supplementary Table S3) for 30 min at 4°C. After several washes, cells were resuspended in PBS and analyzed on an Accuri C6 Flow Cytometer System (BD Biosciences). Fluorescent isotype antibodies (Supplementary Table S3) were used at the same concentrations as controls.

Supplementary Table S1: Number of GFP-expressing iNSCs in the brain, kidney, lung and blood at 1 and 7 days after CHI (n=3)

	CHI 1	CHI 7
Brain	92.33 ± 9.61 (per mm^2)	78.67 ± 7.64 (per mm^2)
Kidney	80.33 ± 7.77 (per mm^2)	59.00 ± 6.24 (per mm^2) *
Lung	73.67 ± 7.77 (per mm^2)	55.00 ± 5.57 (per mm^2) *
Blood	1077.33 ± 117.82 (per μl)	185.33 ± 19.86 (per μl) ***

Note: *P<0.05 versus CHI 1; ***P<0.001 versus CHI 1.

Supplementary Table S2: Neurological severity score (NSS)

Task	Points
Inability to exit a 30-cm diameter circle (for 3 min)	1
Presence of monoparesis or hemiparesis	1
Inability to walk straight	1
Loss of startle reflex	1
Loss of seeking behavior	1
Inability to balance on a 0.7-cm wide beam (for 10 s)	1
Inability to balance on a round stick of 0.5 cm diameter (for 10 s)	1
Inability to cross a 30-cm long beam of 3 cm width	1
Inability to cross a 30-cm long beam of 2 cm width	1
Inability to cross a 30-cm long beam of 1 cm width	1
Maximum total	10

Neurological impairment post-CHI was assessed by neurological severity score (NSS). One point was given for a tested reflex’s absence or for failing to perform an individual task.

Supplementary Table S3: Antibodies were used in this study

Specificity	Host	Detection	Concentration	Application	Origin
C3d	Goat	Brain/Kidney/Lung	5 µg/ml	IF/WB	R&D Systems
C5b-9	Rabbit	Brain/Kidney/Lung/INSC	2 µg/ml	IF/WB	Abcam
NeuN	Mouse	Brain/Neuron	5 µg/ml	IF	Millipore
Map2	Chicken	Brain	2 µg/ml	IF	Abcam
Crry	Rat	Brain/Kidney/Lung/INSC	5 µg/ml	IF/FC/WB	BD Biosciences
C3	Rat	INSC	5 µg/ml	IF/FC	Abcam
GFAP	Rabbit	Astrocyte	0.5 µl/ml	IF	CST
Olig2	Rabbit	Oligodendrocyte	1 µg/ml	IF	Millipore
MPO	Rabbit	Neutrophil	1 µg/ml	IF	Abcam
Active Caspase-3	Rabbit	Brain/Kidney/Lung	1 µg/ml	IF/WB	Abcam
Bax	Rabbit	Brain/Kidney/Lung	1:1000	IF/WB	CST
Iba1	Goat	Microglia/Macrophage	1 µg/ml	IF	Abcam
Goat IgG	Alexa Fluor® 555 Donkey	C3d/Iba1	2 µg/ml	IF	Life Tech
Rabbit IgG	Alexa Fluor® 555 Goat	C5b-9/GFAP/Olig2/MPO/ Active Caspase-3/Bax	2 µg/ml	IF	Life Tech
Mouse IgG	Alexa Fluor® 633 Goat	NeuN	2 µg/ml	IF	Life Tech
Chicken IgG	Alexa Fluor® 633 Goat	Map2	2 µg/ml	IF	Life Tech
Rat IgG	Alexa Fluor® 633 Goat	Crry/C3	2 µg/ml	IF	Life Tech
C9	Rabbit	Brain/Kidney/Lung	1 µg/ml	WB	Abcam
GAPDH	Rabbit	Brain/Kidney/Lung/INSC	0.2 µg/ml	WB	Santa Cruz
Goat IgG	Rabbit	C3d	0.08 µg/ml	WB	ZSGB-BIO
Rabbit IgG	Goat	C5b-9/C9/Active Caspase-3/Bax/GAPDH	0.08 µg/ml	WB	ZSGB-BIO
Rat IgG	Goat	Crry	0.08 µg/ml	WB	ZSGB-BIO

Rat IgG2α, κ Isotype Control		5 µg/ml	FC	BD Biosciences
APC Goat Anti-Rat IgG	Crry/C3/Rat IgG2α, κ Isotype Control	2 µg/ml	FC	BD Biosciences
CD46 Rabbit	INSC/NSC	10 µg/ml	FC	Abcam
Rabbit IgG, polyclonal Isotype Control		10 µg/ml	FC	Abcam
APC Goat Anti-Rabbit IgG	CD46/Rabbit IgG, polyclonal Isotype Control	5 µg/ml	FC	Thermo Scientific
PE Anti-Mouse CD59a		2 µg/ml	FC	Biolegend
PE Mouse IgG1, κ Isotype Control		2 µg/ml	FC	Biolegend
PE Hamster Anti-Mouse CD55		2 µg/ml	FC	BD Biosciences
PE Hamster IgG3, λ1 Isotype Control		2 µg/ml	FC	BD Biosciences

IF: Immunofluorescence; WB: Western blot; FC: Flow cytometry

R&D Systems, Minneapolis, MN, USA; Abcam, Cambridge, MA, USA; Millipore, Bedford, MA, USA; BD Biosciences, San Jose, CA, USA; CST, Beverly, MA, USA; Life Tech, Gaithersburg, MD, USA; Santa Cruz, Santa Cruz, CA, USA; ZSGB-BIO, Beijing, China; Thermo Scientific, Hudson, NH, USA; Biolegend, San Diego, CA, USA

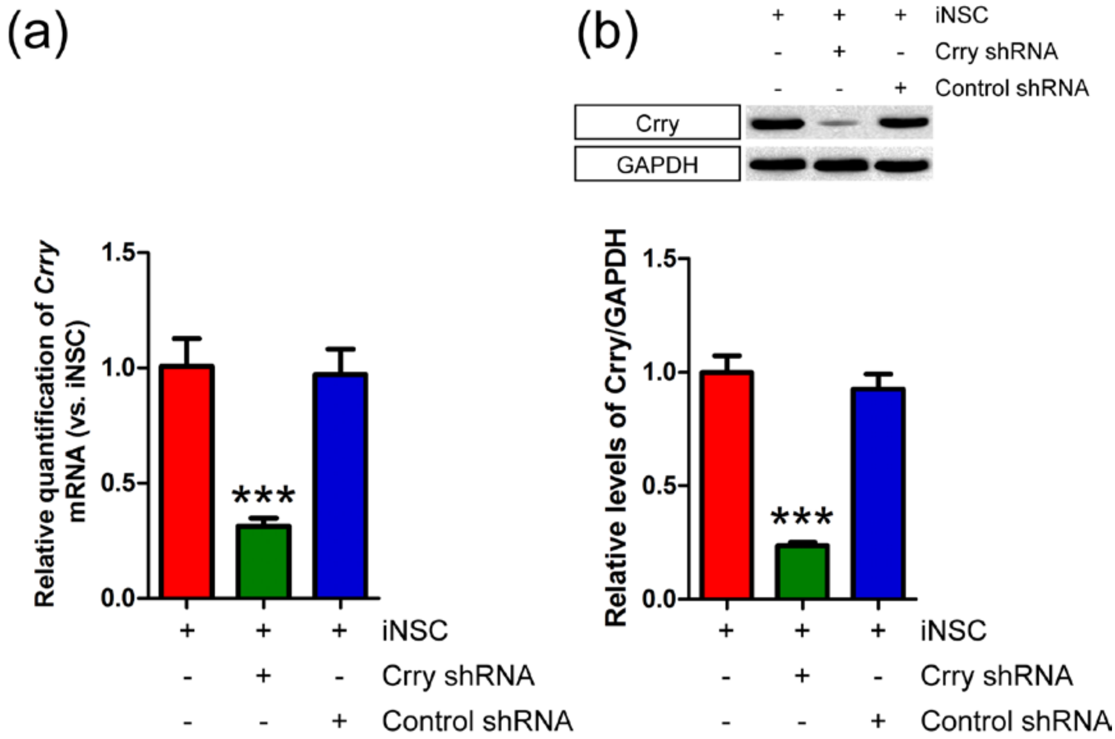

Supplementary Figure S1. Expression level of Crry in iNSCs was assayed by RT-QPCR and western blot. (**a**) Histograms indicated *Crry* gene expression in iNSCs among the iNSC, iNSC (Crry shRNA) and iNSC (Control shRNA) groups (n=3/group; ****P*<0.001). (**b**) Representative immunoblots depicted Crry protein expression in iNSCs among these three groups. Histograms showed the relative level of Crry in iNSCs among these three groups (n=6/group; ****P*<0.001).

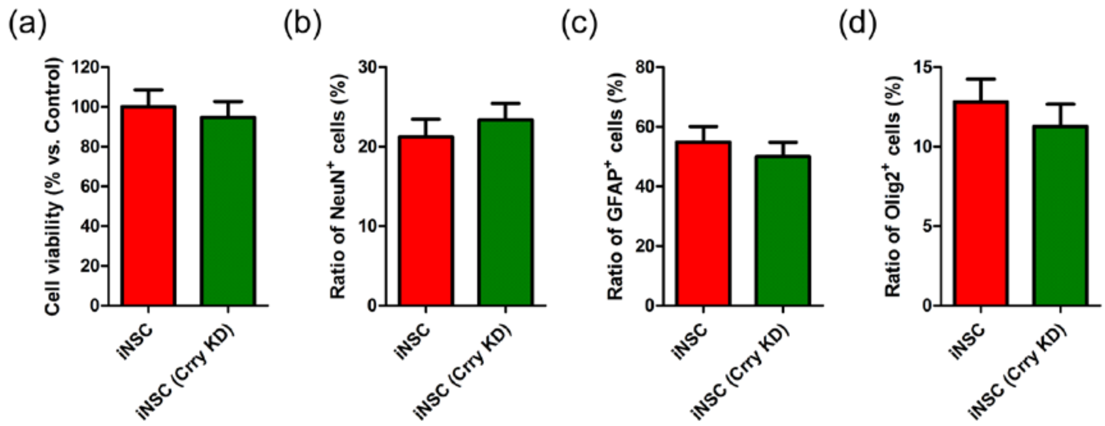

Supplementary Figure S2. Cell viability assay and differentiation. (**a**) The viability of iNSCs and iNSCs (Crry KD) without CHI mouse serum treatment was detected by MTT assay (n=3/group). (**b-d**) For differentiation, iNSCs and iNSCs (Crry KD) were dissociated using accutase and seeded on glass cover slips coated with poly-l-lysine on a 24-well plate ($5x10^4$ cells per well) in differentiation medium (DMEM/F12 containing 2% B27 supplements and 0.5% fetal bovine serum) for several days. Histograms showed the levels of $NeuN^+$ neurons (**b**), $GFAP^+$ astrocytes (**c**), and $Olig2^+$ oligodendrocytes (**d**) derived from iNSCs and iNSCs (Crry KD) (n=3/group).

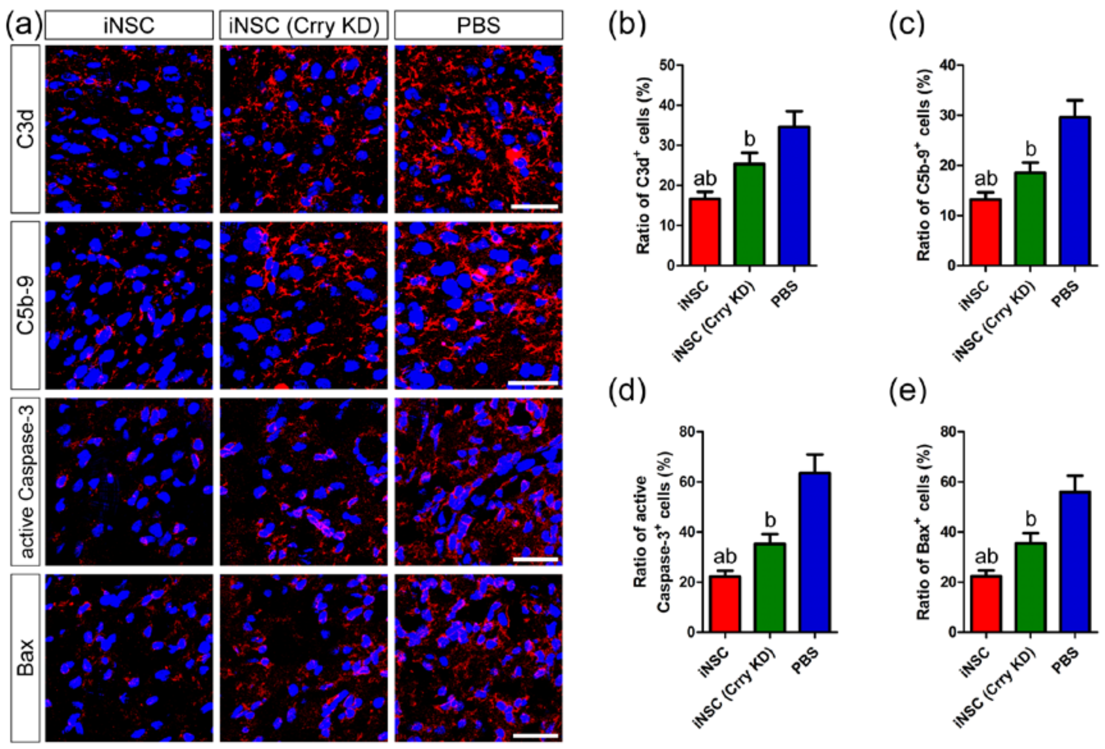

Supplementary Figure S3. INSC grafts modulated complement activation in CHI mice via Crry expression. (**a**) Representative staining for C3d$^+$ (red), C5b-9$^+$ (red), active Caspase-3$^+$ (red), and Bax$^+$ (red) cells depicted the distribution of C3d$^+$, C5b-9$^+$, active Caspase-3$^+$ and Bax$^+$ cells in the injured cerebral cortex among the iNSC, iNSC (Crry KD) and PBS groups on day 7 post-CHI. Nuclei were counterstained with DAPI (blue). (**b-e**) Histograms indicated the numbers of C3d$^+$ (**b**), C5b-9$^+$ (**c**), active Caspase-3$^+$ (**d**), and Bax$^+$ (**e**) cells in the injured cerebral cortex among the three groups on day 7 post-CHI (n=3/group; (**a**) P<0.05 versus iNSC (Crry KD) group; (**b**) P<0.05 versus PBS group). Scale bar=50 μm.

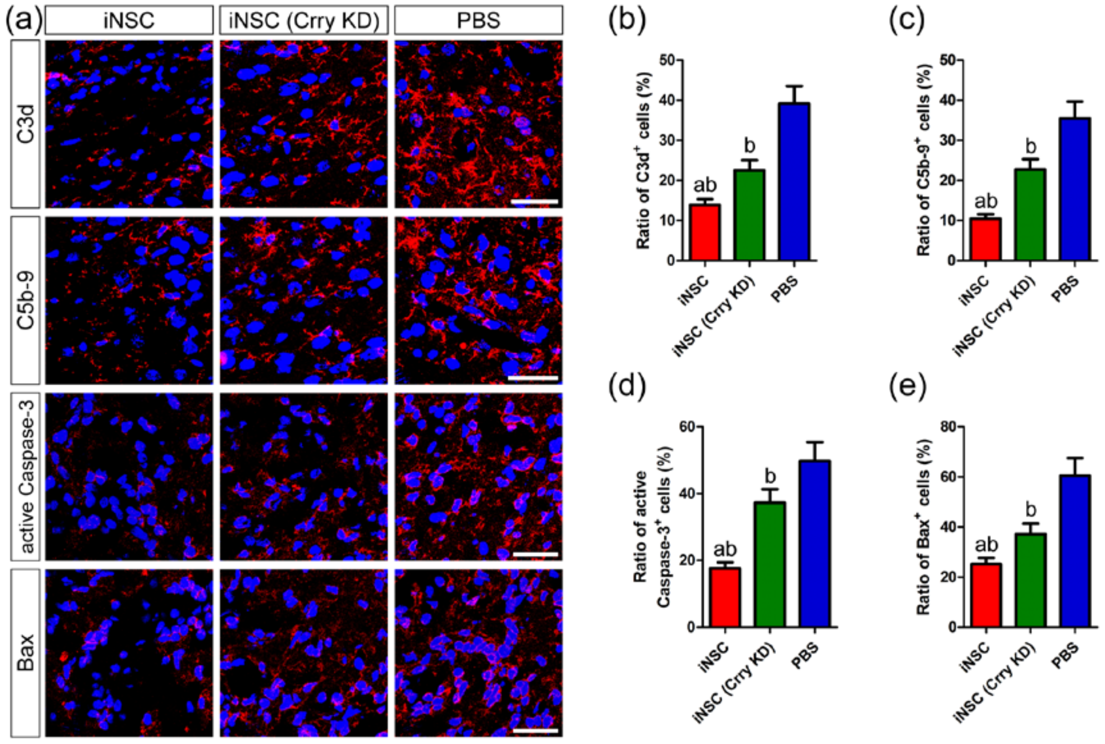

Supplementary Figure S4. INSC grafts modulated complement activation in CHI mice via Crry expression. (**a**) Representative staining for C3d$^+$ (red), C5b-9$^+$ (red), active Caspase-3$^+$ (red), and Bax$^+$ (red) cells depicted the distribution of C3d$^+$, C5b-9$^+$, active Caspase-3$^+$ and Bax$^+$ cells in the glomeruli of the kidney among the iNSC, iNSC (Crry KD) and PBS groups on day 7 post-CHI. Nuclei were counterstained with DAPI (blue). (**b-e**) Histograms indicated the numbers of C3d$^+$ (**b**), C5b-9$^+$ (**c**), active Caspase-3$^+$ (**d**), and Bax$^+$ (**e**) cells in the glomeruli of the kidney among the three groups on day 7 post-CHI (n=3/group; (**a**) $P<0.05$ versus iNSC (Crry KD) group; (**b**) $P<0.05$ versus PBS group). Scale bar=50 μm.

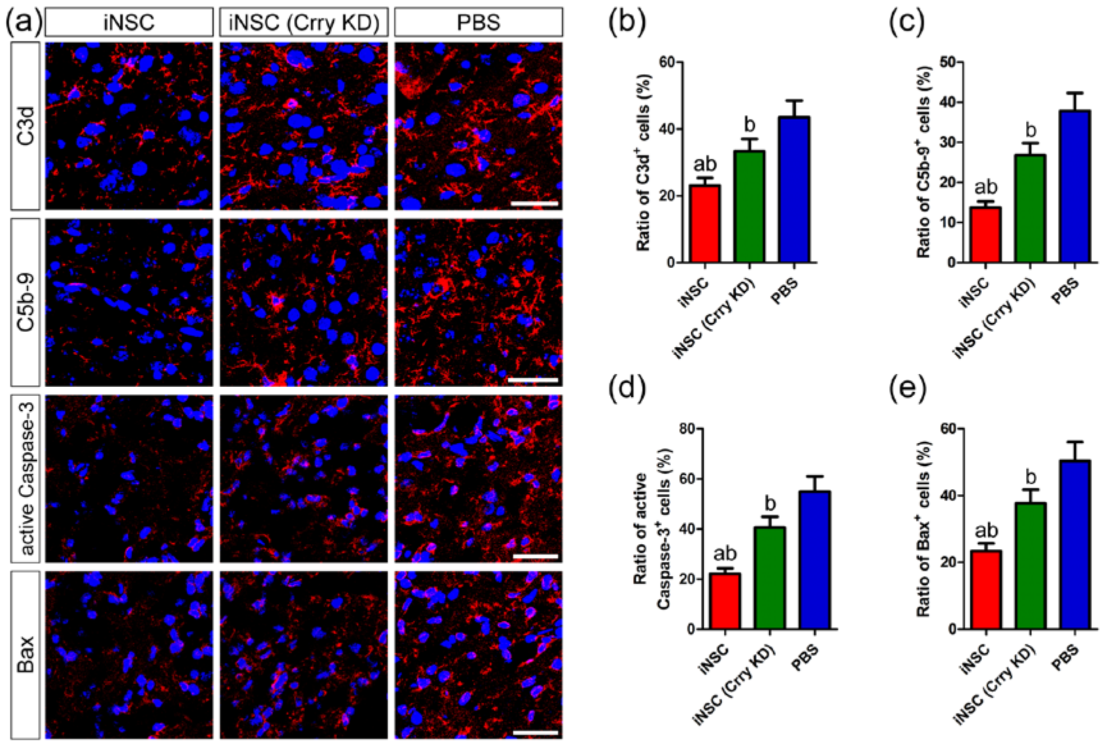

Supplementary Figure S5. INSC grafts modulated complement activation in CHI mice via Crry expression. (**a**) Representative staining for C3d^{+} (red), C5b-9^{+} (red), active Caspase-3^{+} (red), and Bax^{+} (red) cells depicted the distribution of C3d^{+}, C5b-9^{+}, active Caspase-3^{+} and Bax^{+} cells in the alveoli of the lung among the iNSC, iNSC (Crry KD) and PBS groups on day 7 post-CHI. Nuclei were counterstained with DAPI (blue). (**b-e**) Histograms indicated the numbers of C3d^{+} (**b**), C5b-9^{+} (**c**), active Caspase-3^{+} (**d**), and Bax^{+} (**e**) cells in the alveoli of the lung among the three groups on day 7 post-CHI (n=3/group; (**a**) $P<0.05$ versus iNSC (Crry KD) group; (**b**) $P<0.05$ versus PBS group). Scale bar=50 μm.

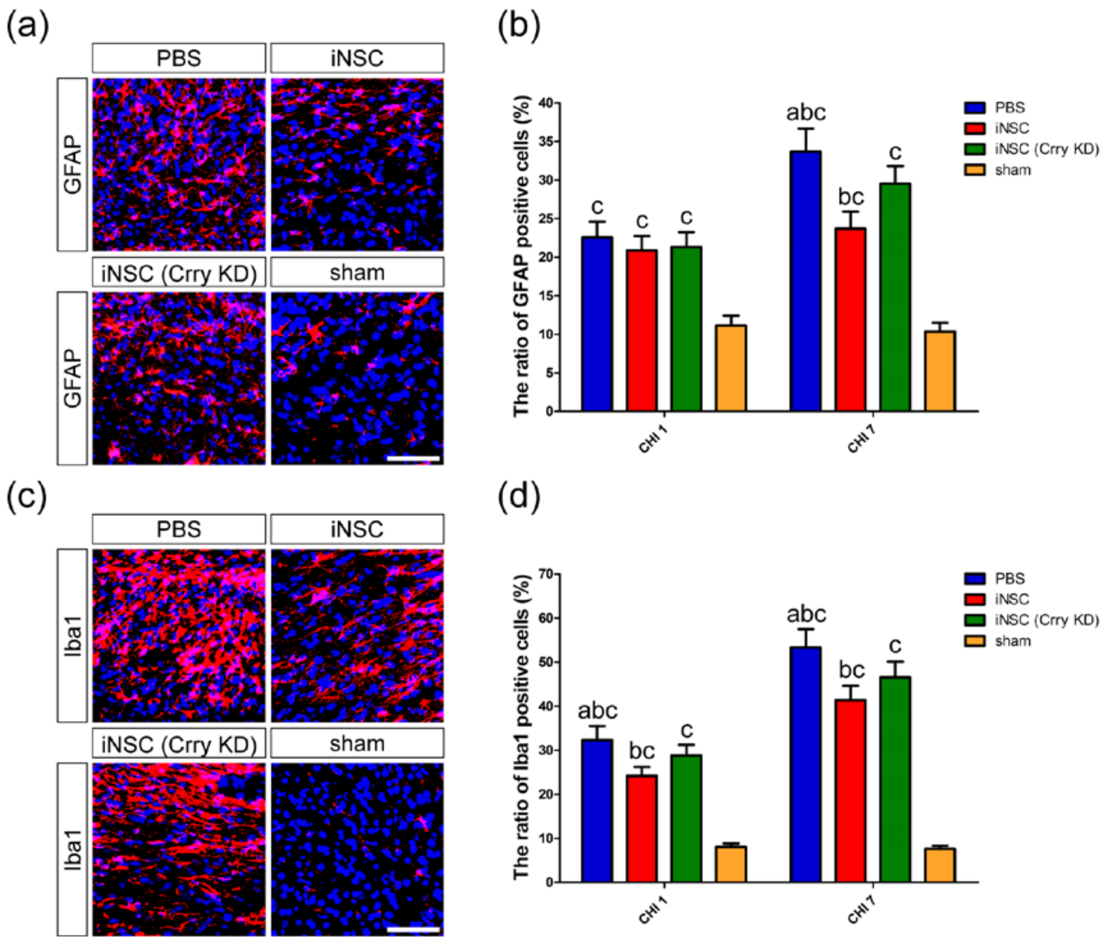

Supplementary Figure S6. INSC grafts inhibited reactive astrogliosis and reduced microglia/macrophage infiltration in CHI mice via Crry expression. (**a**) Representative staining for $GFAP^{+}$ (red) cells depicted the distribution of $GFAP^{+}$ astrocytes in the injured cortex among the four groups on day 7 post-CHI. (**b**) Histograms showed the numbers of $GFAP^{+}$ cells in the injured cortex among the four groups on day 1 and 7 post-CHI (n=6/group; (**a**) $P<0.05$ versus iNSC group; (**b**) $P<0.05$ versus iNSC (Crry KD) group; (**c**) $P<0.05$ versus sham group). (**c**) Representative staining for $Iba1^{+}$ (red) cells depicted the distribution of $Iba1^{+}$ microglia/macrophages in the injured cortex among the four groups on day 7 post-CHI. (**d**) Histograms showed the numbers of $Iba1^{+}$ cells in the injured cortex among the four groups on day 1 and 7 post-CHI (n=6/group; (**a**) $P<0.05$ versus iNSC group; (**b**) $P<0.05$ versus iNSC (Crry KD) group; (**c**) $P<0.05$ versus sham group). Scale bar=100 μm.
